# Pedicled Fillet of Leg Flap for Extensive Pressure Sore Coverage

**Published:** 2009-10-27

**Authors:** Shareef Jandali, David W. Low

**Affiliations:** Division of Plastic Surgery, University of Pennsylvania Health System, Philadelphia

## Abstract

**Objective:** Multiple large decubitus ulcers present a reconstructive challenge to the plastic surgeon. When stage IV pressure sores become recurrent or extensive, traditional flaps either have already been exhausted or would not be sufficient to cover the defect. **Methods:** A retrospective review was performed on all paraplegic patients who had chronic, extensive, and stage IV decubitus ulcers, and underwent reconstruction using a pedicled continuous musculocutaneous flap of the entire leg between 1998 and 2007. The extent and size of the debrided pressure sores, number of previous flap reconstructions, intraoperative blood loss, postoperative complications, and years of follow-up were all recorded. A description of the operative technique is also given. **Results:** Four patients underwent a total leg fillet flap in the study period, with follow-up ranging from 2 to 7 years. Indications included extensive and bilateral trochanteric, sacral, and ischial pressure sores. Complications included intraoperative blood loss and postoperative heterotopic calcification. **Conclusions:** The total leg fillet flap is a very large and robust flap that offers paraplegic patients coverage of extensive stage IV pressure sores of the trochanteric, sacral, and ischial areas.

Multiple large decubitus ulcers present a reconstructive challenge to the plastic surgeon. They are a recurrent and pervasive problem in patients who are either immobilized or insensate in the lower trunk and extremities. Various local and regional flaps from the buttock and thigh are routinely used to close primary ulcerations of modest size. However, when stage IV pressure sores become recurrent or extensive, these flaps either have already been exhausted or would not be sufficient to cover the defect. Reconstruction is aimed at improving hygiene and quality of life, prevention of osteomyelitis and sepsis, prevention of fluid and protein loss through the wound bed, and prevention of future malignancy (Marjolin's ulcers) in these chronic wounds with sinus tracts. We present a method of reconstruction, using a pedicled continuous musculocutaneous flap of the entire leg to fully cover such defects that was performed on 4 patients from 1998 through 2007.

## METHODS

A retrospective review was performed on all paraplegic patients who had chronic, extensive, and stage IV decubitus ulcers, and underwent reconstruction using a pedicled continuous musculocutaneous flap of the entire leg between 1998 and 2007. The extent and size of the debrided pressure sores, number of previous flap reconstructions, intraoperative blood loss, postoperative complications, and years of follow-up were all recorded.

**Figure 1:**
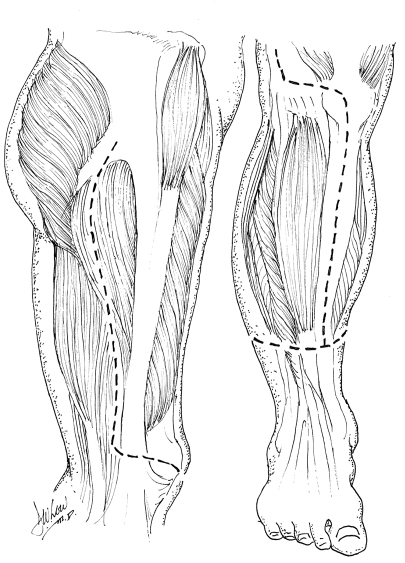
Illustration of incisions for pedicled fillet of leg flap.

**Figure 2:**
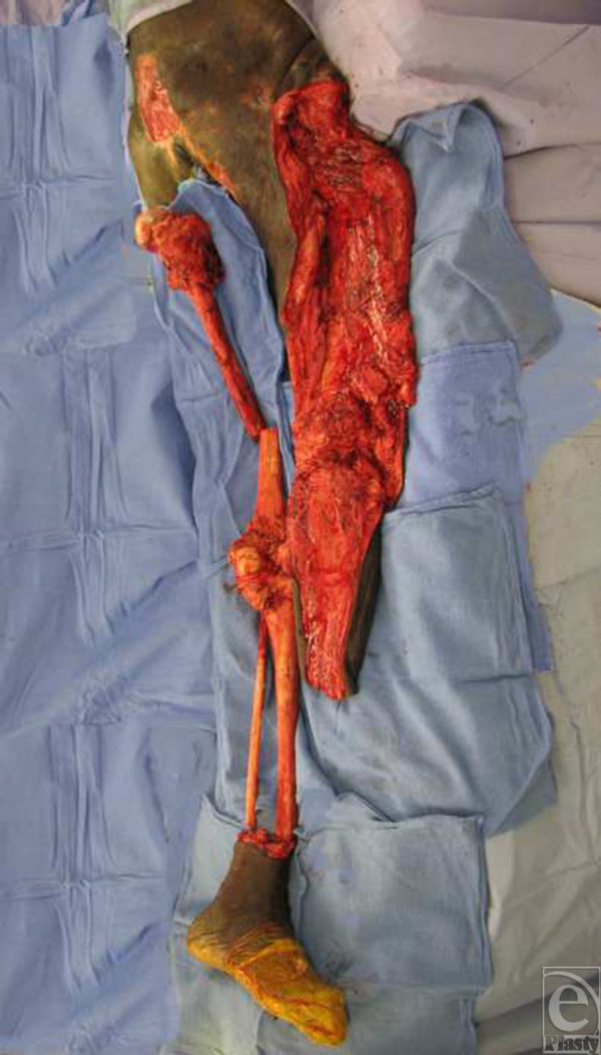
Pedicled leg flap void of any bony support.

**Figure 3:**
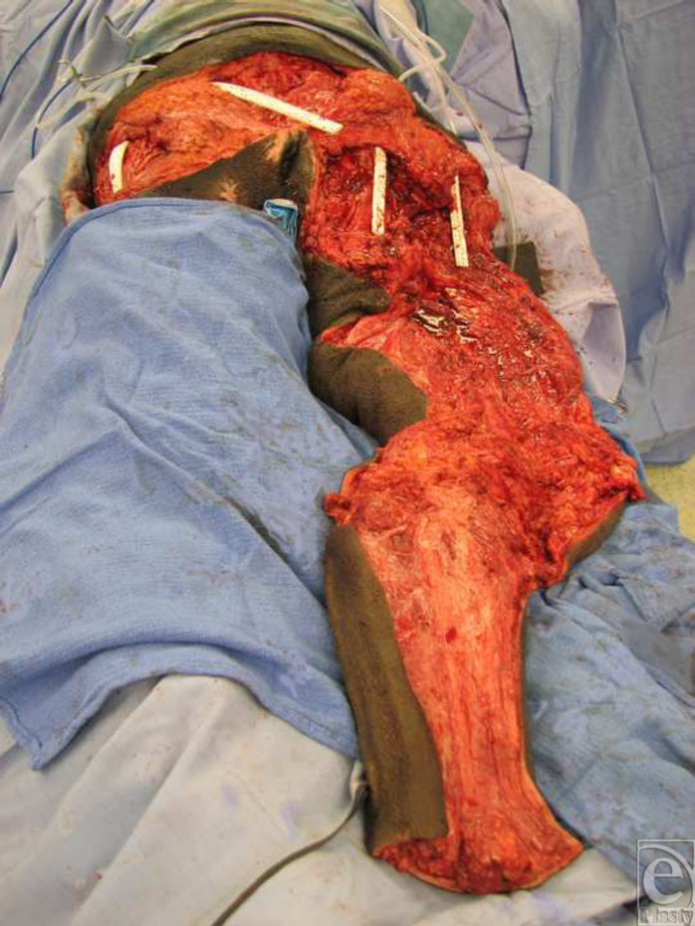
Drain placement prior to insetting of leg flap.

**Figure 4:**
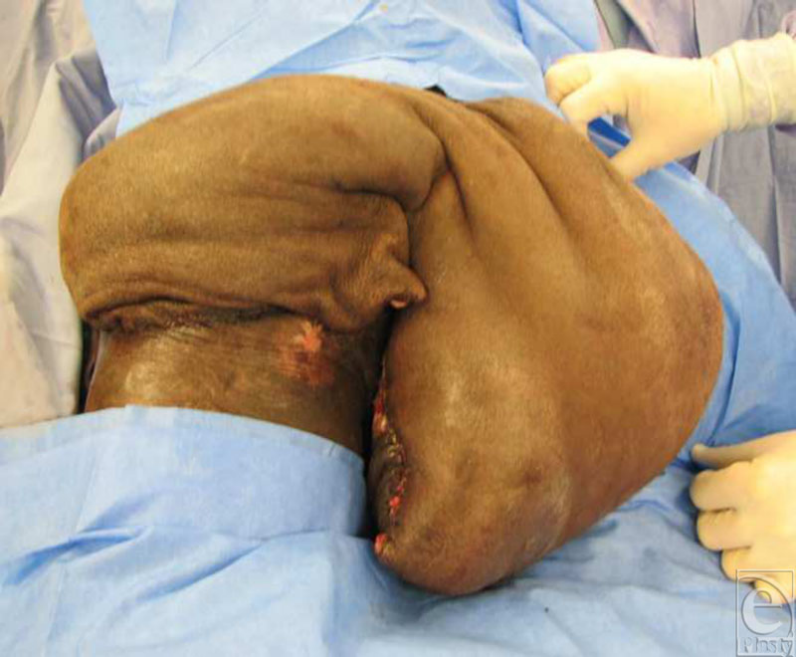
Final coverage of extensive defect after insetting flap.

All patients selected were highly motivated to be compliant with future wound care and prevention of recurrent pressure sores. They had all been serially debrided by either the plastic surgery team or the orthopedic team at our institution. At the time of flap coverage, the multiple pressure sores were debrided a final time, connected into a single wound, and pulse-lavaged. The incision along the thigh was made laterally between the quadriceps muscles and the hamstring muscles so as to avoid dissection of any major muscle groups and rapidly expose the femur. Transection of the mid-femur early in the procedure permitted simultaneous manipulation of both hip and knee joints, thus facilitating a 2-team dissection. The hip was first disarticulated before removing the proximal femur from the leg. The lateral thigh incision was carried transversely across the patella and then along the bare area of the tibia (Fig 1). Using a periosteal elevator, the tibia was exposed in a subperiosteal fashion. Care was taken to avoid injury to the interosseous membrane to preserve flow to the anterior tibial vessels. To facilitate dissection, the foot was amputated just above the ankle by making a circumferential incision and then identifying and ligating the posterior tibial, anterior tibial, and peroneal vessels. After dissection of the tibia, the knee joint was entered and the meniscus was removed from the surrounding soft tissues of the knee capsule. The patella was dissected away from the quadriceps and patellar tendons. The tibia was then removed after separating it from the head of the fibula. The fibula was approached through a submuscular tunnel where both the fibula head and the distal end of the fibula were dissected out in a subperiosteal fashion predominantly using an elevator. The fibula was then pulled through the muscle tunnel so that the leg was completely void of any bony support (Fig 2). The distal end of the leg flap was split into a bilobed flap if needed in order to ensure proper wound coverage. Multiple drains were placed to adequately drain any potential space underneath the flap (Fig 3). Closure was obtained with interrupted deep absorbable sutures between parts of the muscle fascia and the deep bed of the ulcer, followed by a standard skin closure (Fig 4). No attempts were made to innervate these flaps with sensory input from above the level of the spinal cord injury. All patients were kept on an air-fluidized bed for the remainder of their hospital stay and were seen by physical and occupational therapy.

## RESULTS

### Case 1

The patient is a 25-year-old woman with a history of paraplegia secondary to transverse myelitis, complicated by scoliosis and chronic vertebral osteomyelitis, who had undergone multiple previous orthopedic procedures for her spine and 2 pedicled flaps for her pressure sores. She had undergone left hip disarticulation and excision of the proximal femur for an extensive hip pressure sore. She had open wounds on the left side of the ischium and along the sacrum, which after debridement measured about 600 cm^2^, and were reconstructed with a left total leg fillet flap. She lost an estimated 1 L of blood and got transfused with 2 units of packed red blood cells. The flap healed well without complication with follow-up lasting 7 years.

### Case 2

The patient is a 60-year-old man with a history of T4 paraplegia for 21 years from a boating accident. He had not undergone any previous flap surgeries for coverage of his pressure sores. He had exposure of his right posterior iliac wing and the posterior portion of his right hip along with small pressure sores on the left ischium. Three weeks after a colostomy, the patient underwent debridement of all of his wounds, resulting in a defect that measured about 650 cm^2^, requiring a right total leg fillet flap for coverage. He lost an estimated 1 L of blood and got transfused with 2 units of packed red blood cells. The flap healed well without complication with follow-up lasting 6 years.

### Case 3

The patient is a 20-year-old woman with a history of spina bifida, paraplegia, and 2 previous flap reconstructions for pressure sores of her right ischium. She still had a large right ischial pressure sore. She underwent a right total leg fillet flap for coverage of a debrided surface area of 550 cm^2^. She lost an estimated 500 mL of blood and got transfused with 1 unit of packed red blood cells. At her 3-month follow-up, she was found to have some breakdown of the distal end of the flap overlying the ischium without bone exposure and was taken back to the operating room for revision of the flap. Within 4 years of follow-up after the revision, she had no further flap complications.

### Case 4

The patient is a 48-year-old man with a history of paraplegia who developed right trochanteric, sacral, and bilateral ischial pressure sores. He had previously undergone one flap surgery, which was a left gluteal V-Y advancement flap to try to cover a left ischial pressure sore. This patient underwent a right total leg fillet flap for a defect that measured about 800 cm^2^. He lost an estimated 1 L of blood and got transfused with 2 units of packed red blood cells. The flap remained extremely robust postoperatively but a large portion of the undersurface did not adhere to the bed of the pressure sore. At 2 areas where there was breakdown of the wound edges, negative pressure wound therapy was applied by using the VAC® System (Vacuum Assisted Closure) (Kinetic Concepts, Inc, San Antonio, Tex) to try to close the space underneath the flap. The space did not close but never became infected and the health of the flap was never compromised. Four months later, the patient was found to have osteonecrosis of his left hip. While in the operating room for disarticulation and debridement of his left hip, our team removed some heterotopic ossification from the distal part of the flap before reinsetting it. He has had no further flap complications in the 2 years of follow-up since this revision.

## DISCUSSION

A fillet is defined as a boneless cut of meat or fish. The concept of a fillet flap is widely accepted in reconstructive plastic surgery for amputation defects of the extremities, pelvic reconstruction, coverage of exposed hardware in severe spina bifida cases, and coverage of multiple extensive pressure sores. The fillet flap can be either a free flap or a pedicled flap. In 1956, Georgiade et al^1^ first described the use of a total thigh flap for large trochanteric ulcers. Reconstruction of pressure sores has also been reported using a pedicled, filleted, split total thigh flap.^2^ Berkas et al^3^ reported on a similar technique but used soft tissue from below the knee (5 cm past the knee) to gain additional flap length for coverage of sacral defects.

As far as we know, the use of an entire leg continuous pedicled fillet flap has not been reported for coverage of multiple, large pressure sores. We have found that when the defects are extensive, the entire length of the leg must be used as a flap to completely reach and cover the opposite trochanteric region. The use of a 2-component pedicled upper and lower leg fillet flap was reported for use in reconstruction of an extensive hemipelvectomy defect.^4^ Only the popliteal vessels connected the upper and lower components in this report.^4^ In our cases, the skin bridge around the knee was preserved and used in the reconstruction.

The advent of microsurgery has also led to the use of the soft tissue of the leg as a free flap for reconstruction of defects. Coverage of extensive pressure sores has been reported using a filleted lower leg myocutaneous free flap.^5^ Pelvic reconstruction after hemipelvectomy has been performed using this same flap from the amputated lower extremity.^6^ In addition, radical upper and lower limb amputations have been reconstructed using a free fillet extremity flap from the same limb.^7^ In our patient population, there was no need to use a free flap since a pedicled leg flap easily reached all sites that needed coverage.

In our small series, all patients tolerated the procedure well and recovered without any adverse events. The 4 patients each lost between 500 and 1000 mL of blood in the operating room and required transfusion of 1 to 2 units of packed red blood cells. Ischemic flap necrosis did not occur in any of the patients, although there was further pressure necrosis of the flap in one patient. One patient developed heterotopic ossification of the distal aspect of the flap, which was excised during a subsequent procedure. Leg amputation did not alter the ability of the patients to transfer out of bed or to wheelchairs. Subjectively, most found mobility to be more facile since they did not have the excess weight of their nonfunctional leg. In addition, they all worked with physical and occupational therapy after their surgeries.

## CONCLUSIONS

Multiple large decubitus ulcers present a reconstructive challenge to the plastic surgeon. Extensive stage IV pressure sores in a permanently immobilized patient can be reconstructed using a pedicled continuous musculocutaneous flap of the entire leg. This is a very large and robust flap that offers coverage of these wounds when other options such as local advancement flaps have been exhausted or would be insufficient in size to cover the defects.

No funds were used supporting this work and the authors have no financial interest.
